# Reduced COX-2 Expression in Aged Mice Is Associated With Impaired Fracture Healing

**DOI:** 10.1359/jbmr.081002

**Published:** 2008-10-13

**Authors:** Amish A Naik, Chao Xie, Michael J Zuscik, Paul Kingsley, Edward M Schwarz, Hani Awad, Robert Guldberg, Hicham Drissi, J Edward Puzas, Brendan Boyce, Xinping Zhang, Regis J O'Keefe

**Affiliations:** 1 The Center for Musculoskeletal Research, University of Rochester Rochester, New York, USA; 2 Department of Pediatrics, University of Rochester Rochester, New York, USA; 3 Institute for Bioengineering and Bioscience, Georgia Institute of Technology Atlanta, Georgia, USA; 4 Department of Orthopaedics, University of Connecticut School of Medicine Storrs, Connecticut, USA

**Keywords:** fracture, aging, cyclooxygenases, prostaglandin E2, endochondral ossification

## Abstract

The cellular and molecular events responsible for reduced fracture healing with aging are unknown. Cyclooxygenase 2 (COX-2), the inducible regulator of prostaglandin E_2_ (PGE_2_) synthesis, is critical for normal bone repair. A femoral fracture repair model was used in mice at either 7–9 or 52–56 wk of age, and healing was evaluated by imaging, histology, and gene expression studies. Aging was associated with a decreased rate of chondrogenesis, decreased bone formation, reduced callus vascularization, delayed remodeling, and altered expression of genes involved in repair and remodeling. *COX-2* expression in young mice peaked at 5 days, coinciding with the transition of mesenchymal progenitors to cartilage and the onset of expression of early cartilage markers. In situ hybridization and immunohistochemistry showed that *COX-2* is expressed primarily in early cartilage precursors that co-express *col-2.* *COX-2* expression was reduced by 75% and 65% in fractures from aged mice compared with young mice on days 5 and 7, respectively. Local administration of an EP4 agonist to the fracture repair site in aged mice enhanced the rate of chondrogenesis and bone formation to levels observed in young mice, suggesting that the expression of *COX-2* during the early inflammatory phase of repair regulates critical subsequent events including chondrogenesis, bone formation, and remodeling. The findings suggest that COX-2/EP4 agonists may compensate for deficient molecular signals that result in the reduced fracture healing associated with aging.

## INTRODUCTION

Although increased age has been a known risk factor for a decreased rate of fracture healing for >30 yr, little progress has been made toward understanding the mechanisms involved.(1–3) The rate of bone repair is progressively reduced with aging from the pediatric population to the elderly.(4,5) Delayed healing results in an increased duration of immobilization, increases the risk of joint stiffness, and is associated with increased morbidity.(4) Delayed healing also increases the risk of inadequate fracture alignment.(6) However, the most significant clinical problem is the development of nonunion. Several studies have established that aging results in an overall reduction in union in numerous fractures.(1–4) This population not only has an increased risk of fracture caused by reduced BMD but also has reduced fracture healing potential. Thus, for any given fracture, the morbidity is greater for the aging population.

Several studies have evaluated gene expression during fracture repair and have established differences in the rate of fracture healing and the pattern of gene expression between young and aged animals. Stabilized 6-wk-old and 1-yr-old rat femur fractures had gene expression examined over a 6-wk period. Whereas fracture union was established in young rats 4 wk after fracture, union was consistently absent in aged rats after 6 wk.(7) Fractures in aged rats had reduced expression of *Indian hedgehog* (Ihh) and *BMP-2*.(7) Other studies have established that fractures in aged mice have delayed expression of bone and cartilage matrix genes, such as *col2*, *aggrecan*, and *osteocalcin*.(8) A recent study of nonstabilized fractures in mice at 4 wk, 6 mo, and 18 mo of age showed decreased fracture callus volume, delayed maturation of the cartilage callus, and reduced expression of *col2* and *colX* in aged animals.(9)

The cyclooxygenases have an important function in fracture repair with evidence suggesting a critical role during all stages of healing.(10–20) *COX-2* is transiently expressed in a number of tissues during periods of inflammation.(21,22) Cyclooxygenase-1 (COX-1) and -2 (COX-2) catalyze the rate-limiting step in the conversion to arachidonic acid to prostaglandin H2 and ultimately to the production of prostaglandin E_2_ (PGE_2_). PGE_2_ binds to four receptor subtypes (EP1–EP4). The EP2 receptor (EP2) and EP4 receptor (EP4) are G_s_-protein-coupled receptors that are expressed on mesenchymal stem cells, chondrocytes, and osteoblasts.(21–23) Activation of EP2 and EP4 stimulates the production of cAMP and activation of protein kinase A (PKA) signaling.(24) Prior work from our laboratory has shown that stem cells along the periosteum are essential for bone repair,(10,25) whereas others have established that EP4-mediated signaling is critical for periosteal bone formation. Delivery of PGE_2_ using an Alzet pump along the periosteal surface resulted in abundant periosteal bone formation in wildtype, EP1^−/−^, EP2^−/−^, and EP3^−/−^ mice. In contrast, EP4 ^−/−^ mice had no bone formation.(26) Additionally, PGE_2_ failed to stimulate the production of bone nodules in cultures of bone marrow-derived mesenchymal stem cells (MSCs) from EP4-deficient mice.(26) The development of agonists that target specific EP receptors raises the possibility of targeted therapies to potentially accelerate bone repair.

Previously, we showed that deficiency of COX-2 in a murine model results in a delay in fracture healing.(19) A stabilized mouse femur fracture model was used to determine whether different levels of COX-2 expression could account for the reduced rate of fracture healing observed with aging. Our findings established reduced COX-2 expression in fractures in aged mice. We further showed that the delayed fracture healing observed in aged mice could be rescued with local delivery of an EP4 selective agonist. The findings establish a role for COX-2 expression in the delayed fracture healing observed with aging.

## MATERIALS AND METHODS

### Experimental animals

All animal studies were done in accordance and with approval of the University Committee on Animal Resources. Fifty-two-week-old female C57BL/6J mice were obtained from the National Institutes on Aging (Bethesda, MD, USA). C57BL/6J, 7- to 9-wk female mice were procured from Jackson Laboratories (Bar Harbor, ME, USA). Stock aged animals were rederived from Jackson Laboratory breeders in 1990, 1998, and 2005. This routine practice by the NIA was done to eliminate genetic drift that could have occurred between the two populations of mice.

### Femur fracture model

Mice received anesthesia using ketamine and xylazine. The skin and underlying soft tissues over the left knee was incised lateral to the patellar tendon. The tendon was displaced medially, and a small hole was drilled into the distal femur using a 26-gauge needle. A stylus pin from a 25G Quincke Type spinal needle (BD Medical Systems, Franklin Lakes, NJ, USA) was inserted into the intramedullary canal and clipped. The wound was sutured closed. Fractures were created using a three-point bending Einhorn device as previously described.(27) Mice were given six subcutaneous injections of 2 mg/kg Buprenorphine (Abbot Laboratories, Abbott Park, IL, USA) every 12 h to control pain. A Faxitron system (Faxitron X-ray, Wheeling, IL, USA) was used to take X-ray images at the time of surgery, 1-week intervals, and at the time of death.

### Histology and analysis

Mice were killed at 3, 5, 7, 10, 14, 18, 21, 25, 30, or 35 days after fracture. A normal mid-diaphysis femoral bone segment was used as a nonfractured day 0 control. Femurs were disarticulated from the hip and trimmed to remove excess muscle and skin. Specimens were stored in 10% neutral buffered formalin for 2 days. The tissues were infiltrated and embedded in paraffin. Alcian blue and orange G along with TRACP staining was done as previously described.(19,20,28) Histomorphometric analysis (*n* = 4 animals per group) was done using a standardized eyepiece grid to measure tissue areas within the fracture callus. Samples were cut at four levels spanning ∼120 μm through the callus, with 30 μm between each level. Each cross-hatch was categorized as not callus (not counted), callus (quantified), and a specific tissue type. A total area of the external callus and areas of individual tissue types such as new bone (mineralized tissue), total cartilage, immature proliferative cartilage, hypertrophic cartilage, and mesenchyme were quantified. Bone was defined as areas of new woven bone. Cartilage was defined as tissues staining blue for proteoglycan. Hypertrophic cartilage was clearly defined by cellular morphology, and other nonhypertrophic areas of cartilage were considered immature cartilage. Finally, mesenchyme was defined as areas containing spindle-shaped fibroblasts without Alcian blue staining. Cortical bone was excluded from the histomorphometric analysis. The target tissue area was divided over the area of the external callus.

In the rescue experiment, aged mice were treated with either vehicle or a nonprostanoid EP4 selective agonist, CP432 (Pfizer, Groton, CT, USA). CP734432 (CP73) was freshly prepared in normal saline containing 3% ethanol. CP73 (100 μl) was injected at the fracture site twice daily for a total daily dose of 20 mg/kg/d.

### Quantitative real-time PCR

The fracture callus and 1 mm of normal bone margin was carefully excised using a scalpel. These samples were frozen in liquid N_2_, pulverized using a nitrogen-cooled mortar and pestle apparatus (Bel-Art, Scienceware, Pequannock, NJ, USA), and purified for total RNA using the TRIzol system (Invitrogen, Carlsbad, CA, USA).(29) The concentration of stock RNA was determined using a spectrophotometer. cDNA was synthesized from 0.5 μg of RNA per callus using a commercial first-strand cDNA synthesis kit (Invitrogen, Carlsbad, CA, USA). cDNA from *n* = 4 mice from each group was pooled. RT-PCR analyses were performed using murine specific primers for *col2a1, colX, osteocalcin, BMP-2* and -*4, COX-2, RANKL,* and *OPG* expression (see Table 1 for specific sequences). The *col2a1* and *colX* mRNAs encode for the collagen matrix proteins type II collagen and type X collagen, respectively. *col2a1* is maximally expressed during chondrocyte proliferation and *colX* peaks during terminal differentiation of chondrocytes. *Osteocalcin* is expressed in fully mature osteoblasts. *BMP-2* and *BMP-4* are genes that encode for two subtypes of the bone morphogenetic proteins that are involved chondrogenesis and bone formation.(30) *RANKL* stimulates osteoclast maturation and bone remodeling, an important phase of fracture repair.(20) Finally, *OPG* mRNA encodes for osteoprotegerin a secreted decoy receptor for RANKL, which serves to regulate osteoclastogenesis.(20) qPCR reaction was performed using SyberGreen (ABgene, Rochester, NY, USA) in a RotorGene real-time PCR machine (Corbett Research, Carlsbad, CA, USA). All genes were compared with a standard *β-actin* control. Data were assessed quantitatively using two-way analysis of covariance comparing relative levels of transcript expression as a function of time and age.

**Table 1:** List of Oligonucleotide Primer Sequences for Real-Time PCR

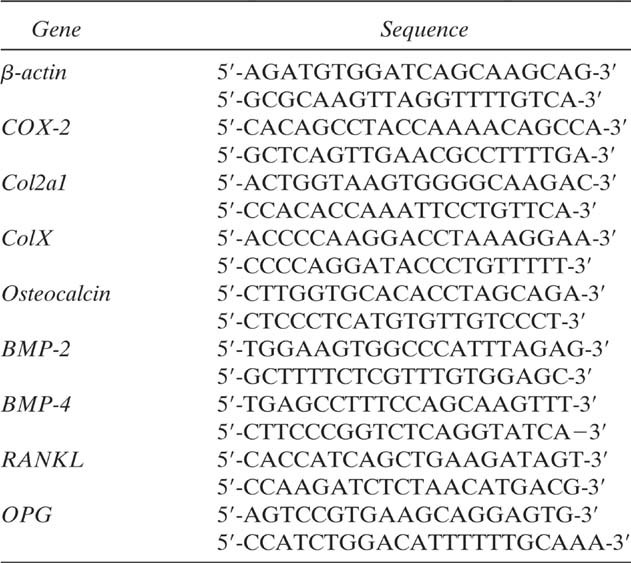

### Immunohistochemistry

Immunohistochemistry was done as previously described.(31) Sections were deparaffinized using xylene and rehydrated using graded alcohols. The endogenous peroxidase was quenched using 3% hydrogen peroxide for 20 min. Nonspecific binding epitopes were blocked using 1:20 normal goat serum. Slides were incubated at 4°C overnight with a 1:200 dilution of mouse primary COX-2 antibody (Cayman Chemical, Ann Arbor, MI, USA). Sections were rewarmed, rinsed in PBS, incubated with a 1:200 goat anti-rabbit secondary antibody, rinsed again in PBS, and finally incubated with a 1:250 horseradish peroxidase (HRP) streptavidin for 30 min. Sections were counterstained with hematoxylin and rinsed. The RANKL goat anti-mouse antibody (Santa Cruz Biotechnologies, Santa Cruz, CA, USA) incubation was done using a 1:20 dilution factor. The immunohistochemical data were qualitatively assessed.

### In situ hybridization

Femurs prepared for in situ hybridization were fixed in 4% paraformaldehyde by intracardiac injection and immersion at 4°C for 3 days. All samples were decalcified in 10% EDTA for 21 days before sectioning. The mouse legs were dissected by disarticulating the femoral head from the hip and immediately immersed in fixative. Excess muscle and pins were carefully removed. Tissues were placed in paraffin and sectioned. In situ hybridization was performed using the mRNAlocator kit (Ambion, Foster City, CA, USA). Proteinase K digestion was done at 37°C for 13 min, and the hybridization reaction was carried out at 55°C overnight. Plasmids for the ^33^P-UTP labeled riboprobes were provided by Jill Helms (*col2*, *colX*, and *osteocalcin*)(30) and Adam Sapirstein (*COX-2*).(32) Sections were observed using both light- and dark-field microscopy and assessed qualitatively.

### μCT

On death, femurs were disarticulated from the hip and fixed in 10% neutral buffered formalin (NBF). For vascular perfusion studies, mice were killed and given serial intracardiac injections of heparinized saline, 10% NBF, and a lead chromate microfil perfusion reagent (Flow Tech, Carver, MA, USA). The whole mice were soaked in 10% NBF overnight at 4°C. The fractured limbs were disarticulated at the hip, and excess soft tissue was removed. Calluses were further fixed in NBF for 2 additional days, and decalcified in 10% EDTA for 21 days. The samples were scanned twice, before and after decalcification, in a VivaCT Scanner (Scanco Medical AG, Bassersdorf, Switzerland) at high resolution with a 12.5-μm voxel size. An integration time of 300 ms, a current of 145 μA, and an energy setting of 55 kV was used. The threshold was chosen using 2D evaluation of several slices in the transverse anatomic plane so that mineralized callus and vascular contrast reagent were identified but surrounding soft tissue was excluded. An average threshold of 250 was optimal and used uniformly for all samples. Next, each sample was contoured around the external callus and along the edge of the cortical bone. All mineralized tissues above threshold between these two boundaries were included. Thus, external soft tissues and cortical bone including the marrow cavity were excluded. Contouring of images was done every 20 axial slices proximally to distally until the callus was not visible. Because all the soft tissue was removed, it was possible to detect the entire callus volume by lowering the threshold. The volume of the mineralized tissue or vascular bed was divided by the volume of the whole external callus. We used an *n* = 5 sample size for each group. Data sets were examined using statistical assessments including ANOVA.

### Statistics

Statistical analysis was performed as previously described.(19) The results were described as mean ± SE. Statistical significance was determined using two-way ANOVA and Student's *t*-tests. These tests determined significance between young/old mice and the values at different time points. *p* < 0.05 was considered significant. Data analysis was performed using GraphPad Prism version 5.0 (GraphPad Software, San Diego, CA, USA).

## RESULTS

### Fractures in aged mice have delayed radiographic healing, decreased bone formation, vascularization, and remodeling

**FIG. 1:**
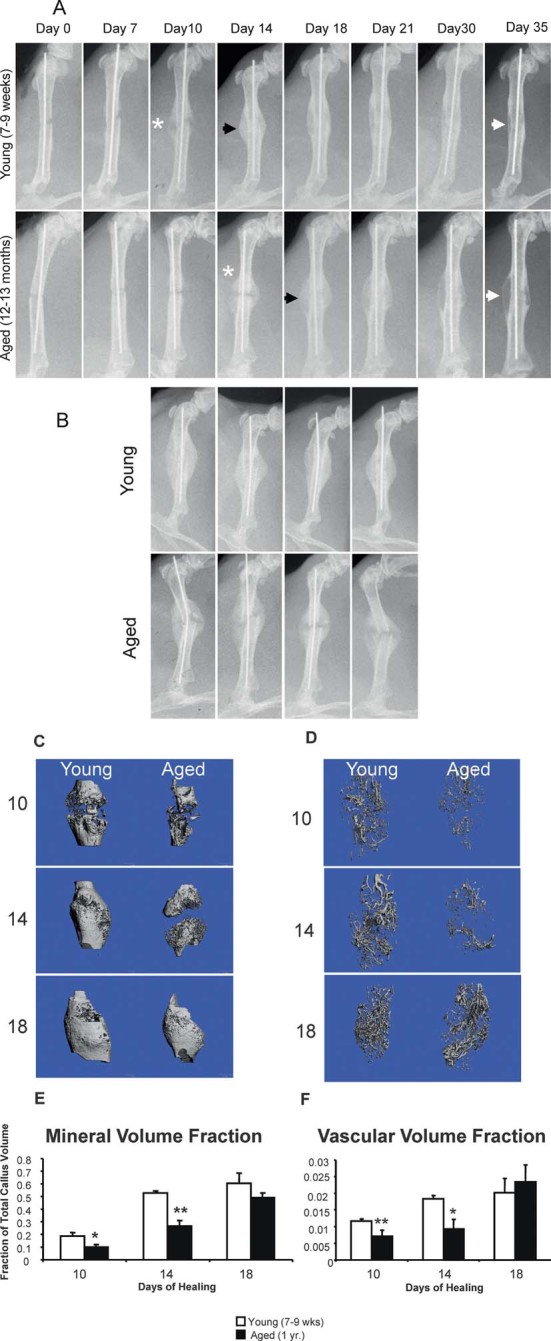
Aged mice have a radiographic delay in fracture healing. Serial radiographs of representative young and aged mice at various times after fracture. Young mice showed evidence of callus mineralization by day 10, which was delayed to day 14 in aged mice (A, *). By day 14 in young mice and day 18 in aged mice, the callus showed union (black arrows). Remodeling was delayed in aged mice compared with young mice (A, white arrows). The delay in fracture repair was consistently observed in day 14 fractures (*n* = 4) (B). Fractures were harvested, and high-resolution μCT scans were performed on young (*n* = 5) and aged (*n* = 5) mice. For vascular analysis, mice were perfused with a lead chromate microfil contrast reagent before decalcification. Representative scans are shown for days 10, 14, and 18 for calcified callus (C) and vascularization (D) for young and aged mice, respectively. Mean calculated mineral (E) and vascular (F) volumes were obtained. Mineral volume analysis showed lower mineral accretion in aged mice, particularly on days 10 and 14 (C and E). On days 10 and 14, aged mice have a significant reduction in the proportion of blood vessels in their calluses compared with young mice (D and F). Statistical comparisons were performed using ANOVA and are denoted by symbols: *p < 0.05 and **p < 0.005.

Radiographs at various time points during fracture healing were obtained (Fig. 1A). Calcified callus and evidence of bone union were observed in the fractures of young mice at days 10 and 14, respectively, but were delayed in fractures from aged mice until 14 and 18 days after fracture (Fig. 1A). The radiographic delay in fracture healing was consistently observed (Fig. 1B; *n* = 4 young and *n* = 4 aged mice). Remodeling also occurred earlier in young mice (Fig. 1A). By 25 days after fracture, clear evidence of remodeling was observed, and radiographic remodeling was nearly complete by 35 days. In fractures from aged mice, limited evidence of radiographic remodeling was observed 35 days after fracture.

μCT was performed on mice harvested at 10, 14, and 18 days after fracture. Previously it has been shown that vascularization is a sensitive marker of the completion of endochondral bone formation and is important in the progression of fracture repair.(33–35) For this reason, the fractures were perfused with lead chromate microfil so that the vascularization within the callus could be determined in fractures in young and aged mice. μCT showed delayed bone formation, fracture union, and vascularization in fractures from aged mice compared with fractures from young mice. Similar to the data observed in radiographs, fracture healing was complete in young mice by 14 days after fracture. In contrast, healing was delayed until 18 days in aged fractures, and callus vascularization was delayed (Figs. 1C and 1D). Quantitative assessment of these parameters showed that mineralization was increased in fractures in young mice compared with aged mice by 1.9- and 2-fold at 10 and 14 days, respectively (Fig. 1E). However, mineralization was similar by 18 days after fracture. Similarly, vascularization was increased 1.7- and 2.0-fold in fractures from young compared with aged mice at 10 and 14 days but reached equivalent values by 18 days after fracture (Fig. 1F). These findings suggest a delay in the completion of endochondral ossification in aging fractures.

### Fractures in aged mice have delayed chondrogenesis and completion of endochondral ossification, impaired bone remodeling, and altered gene expression

**FIG. 2:**
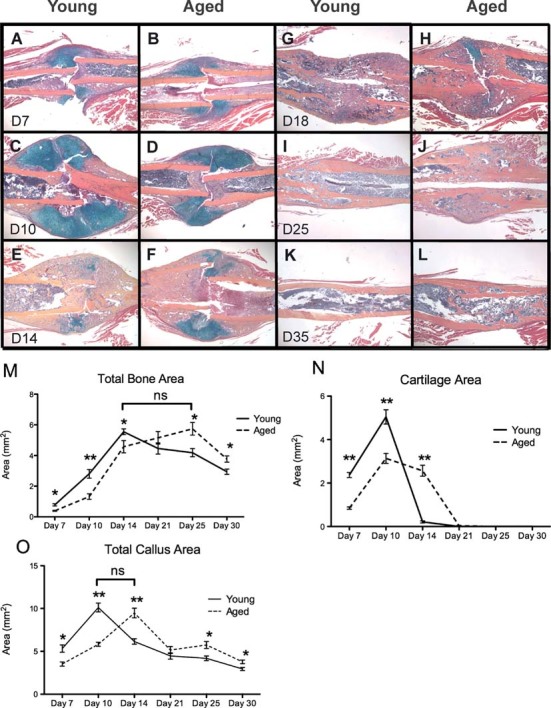
Fractures in aged mice have delayed cartilage and bone formation. Histology sections were prepared from young and aged mice harvested at various times after fracture (A-L). By day 10, calluses were composed of abundant mature cartilage in young mice compared with largely immature cartilage in aged mice. By day 14, most callus tissue consisted of new bone in young animals, whereas aged animals showed a persistence of cartilage and delayed completion of endochondral ossification. On days 18, 25, and 35, young mice had more advanced remodeling compared with aged animals. Histomorphometric analysis of total callus, bone, and cartilage areas were completed in *n* = 4 young and *n* = 4 aged mice, respectively, with three levels analyzed per sample. Histomorphometry showed delayed formation of cartilage, bone and total callus area in aged mice (M-O). Statistical comparisons were performed using ANOVA and significant differences are denoted by symbols: *p < 0.05 and **p < 0.005.

Histology sections of the fractures confirmed a delay in fracture healing and remodeling in aged mice (Fig. 2). Whereas cartilage formation was observed in both young and aged mice on day 7, cartilage undergoes more rapid maturation and endochondral bone formation is complete by day 14 in young mice. In contrast, cartilage is present in aged fractures at 14 days and areas of cartilage remains through 18 days. Histomorphometry was performed at each time point to quantify the amount of mesenchyme, cartilage, and bone present in the sections (Figs. 2M and 2O). Fractures in aged mice had reduced callus formation early and delayed chondrogenesis, bone formation, and remodeling (Figs. 2M and 2O). Total bone area was significantly higher in the fractures of young mice on days 7, 10, and 14 but was lower at days 25 and 30 after remodeling of the fracture. In contrast, aged animals continue to have a net increase in bone area until day 25 (Fig. 2M). In young animals, peak cartilage area is observed at day 10 and is almost entirely replaced by bone by day 14. However, in aged animals, abundant cartilage persists at day 14. Fractures in both young and aged mice have calluses almost entirely composed of bone by day 21 (Fig. 2N). Delayed bone formation and chondrogenesis result in delayed peak total callus area and remodeling in aged animals (Fig. 2O).

Histology showed more rapid remodeling in fractures in young mice (Fig. 2). By 18 days, young mice have evidence of extensive remodeling, and at 25 days, most of the initial woven bone within the callus has been remodeled. By 35 days, the fractures in young mice have essentially undergone complete remodeling and the femur is achieving a morphology similar to the nonfractured bone. These events are markedly delayed in the fracture callus from aged mice. Extensive remodeling is not observed until 25 days, and at 35 days, fracture callus in aged mice remains enlarged and contains areas of woven bone (Fig. 2).

### Expression of genes involved in matrix accumulation, bone formation, and remodeling is altered in fractures from aged mice

**FIG. 3:**
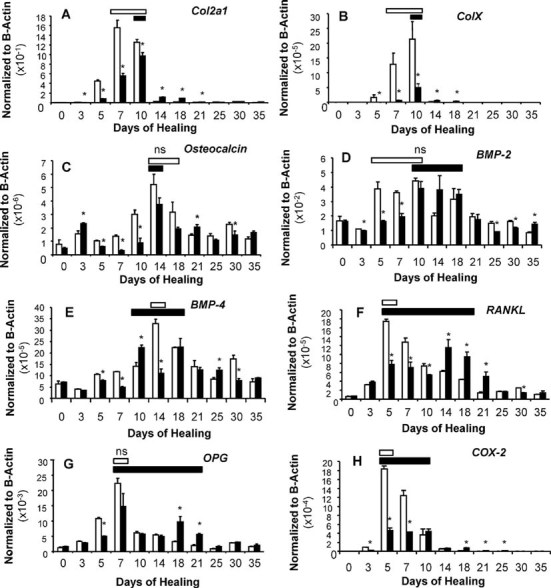
Fracture in aged mice have altered patterns of gene expression during the chondrogenesis, bone formation, and remodeling phases of repair. Total RNA was harvested and pooled from *n* = 4 young and aged mice at various points. Real-time RT-PCR was performed and normalized to *β-actin* expression. Values from young mice are shown in the white bars and aged mice in the black bars. The bars at the top of each figure denote the period of time in which maximal expression of each gene occurred in the fractures from both young (white bar) and aged (black bar) mice. Peak expression of *col2a1*, *COX-2*, *RANKL*, and *OPG* occur in fractures in young mice during the chondrogenic phase of fracture healing between days 0 and 7 (A, F, G, and H). *ColX* and BMP-2 are maximal at day 10 during the peak of endochondral bone formation (B and D). Finally, *osteocalcin* and *bmp-4* gene expression (C and E) are maximal at day 14, consistent with the peak of primary bone formation on the cartilage template. In contrast, fractures have mice have an altered pattern of gene expressions. *COX-2* gene expression was diminished on days 3, 5, and 7 during the phase of chondrogenesis (H). Other genes were delayed, had reduced or delayed maximal expression, or had a broader duration of expression. Statistical comparisons were performed using ANOVA and significance denoted by symbols: *p < 0.05 and **p < 0.005. Peak expression periods are denoted by the bars at the top of the figure. The maximal value measured in young and old mice for each gene is statistically different (*p* < 0.05; A, B, E, F, and H) in all cases except for panels in which “ns” appears with the bars (C, D, and G).

Total RNA was harvested from fractures at various times between 3 and 35 days to examine molecular events underlying the delayed healing observed in aged mice. In the fractures from young mice, *col2a1* appeared earlier, and peak levels were observed by days 7–10 compared with maximal expression at day 10 in aged mice (Fig. 3A). Similarly, maximal expression of the chondrocyte maturation marker, *colX*, occurred from days 7 to 10 in fractures from young mice, whereas aged mice had maximal expression occurring at day 10. Maximal *col2a1* and *colX* expressions were significantly higher in fractures from young mice (Fig. 3B), consistent with the increase in cartilage observed in young mice by histomorphometry. Moreover, both *col2a1* and *colX* expressions disappear in young mice by day 14, signifying completion of the endochondral phase of fracture repair, whereas cartilage matrix gene expression persists until day 21 in aged mice.

Early and late peaks of *osteocalcin* expression were observed (Fig. 3D). An early minor peak occurred at day 3 when initial intramembranous ossification occurs along the periosteal bone surface. *Osteocalcin* levels were slightly higher in fractures from aged mice at this early peak compared with those observed in young mice. However, by 5 through 10 days, *osteocalcin* levels were significantly increased in fractures from young mice. Peak expressions were reached in both young (14–18 days) and aged mice (14 days), and although higher in young mice, the difference was not statistically significant. At 21 days, *osteocalcin* expression was elevated in fractures from aged mice compared with fractures from young mice. Altogether, the osteocalcin expression levels are consistent with a more robust early formation of bone in fractures from young mice, consistent with their earlier completion of endochondral ossification

Expressions of *BMP-2* and *BMP-4* were examined in the fracture calluses, and distinct temporal patterns of expression were observed (Figs. 3E and 3F). In fractures harvested from young mice *BMP-2,* expression was elevated early during the endochondral phase of fracture repair, with peak expressions present between 5 and 10 days, with a subsequent decrease during the bone formation phase of repair. In contrast, *BMP-4* expression was highest at 14 days, corresponding to the peak of osteocalcin expression and bone formation.

*BMP-2* and *BMP-4* had an overlapping pattern of expression in aged mice (Figs. 3E and 3F). *BMP-2* expression was maximal in aging fractures between 10 and 18 days compared with 5–10 days in young mice. Similarly, *BMP-4* expression was elevated between 10 and 18 days in contrast to the single peak of expression that occurred in young mice at 14 days. This is consistent with less distinct cartilage and bone formation phases of fracture healing observed in the aged mice.

Finally, consistent with the diminished bone remodeling observed on radiographs and histological sections, the pattern of *RANKL* and *OPG* expressions was markedly altered in fractures from aged mice (Figs. 3G and 3H). Peak *RANKL* expression occurs surprisingly early in the process of fracture repair and is observed at the onset of chondrogenesis. Young mice had an abrupt peak in *RANKL* expression at 5 days with a gradual decline to basal levels by 21 days, suggesting that the induction of osteoclastogenesis and remodeling is one of the early molecular events to occur in fracture repair. A similar abrupt peak in *OPG* expression was observed in fractures in young mice but occurred at day 7 after fracture and thus immediately followed the induction in *RANKL*. In contrast, fractures in aged mice showed a less brisk but more prolonged elevation of *RANKL* from days 5 to 18 after fracture. Interestingly *OPG* gene expression pattern mirrored that observed with *RANKL*, but with peak levels occurring between 7 days and at 21 days in fractures from aged mice. These findings show that *RANKL* and *OPG* expressions are temporally linked, with maximal *RANKL* expression occurring well before the peak of bone remodeling and immediately followed by an increase in the expression of the decoy receptor, *OPG*.

### COX-2 is altered in fractures in aged mice

*COX-2* has previously been shown to be an important anabolic agent for fracture repair.(18,19,36) In normal bone harvested from the femoral shaft, basal levels of *COX-2* expression was similar in young and aged bone tissue (Supplemental Fig. 1). After fracture, *COX-2* expression was induced early and preceded the onset of chondrogenesis (Fig. 3H). In fractures from young mice, *COX-2* was increased by 3 days and had a sharp peak of maximal expression at 5 days. *COX*-2 levels declined rapidly and returned toward baseline levels by 10–14 days. In fractures from aged mice, the magnitude *COX-2* induction was markedly less and the peak expression was less distinct so that a much lower level of maximal *COX-2* expression was sustained between 5 and 10 days.

**FIG. 4:**
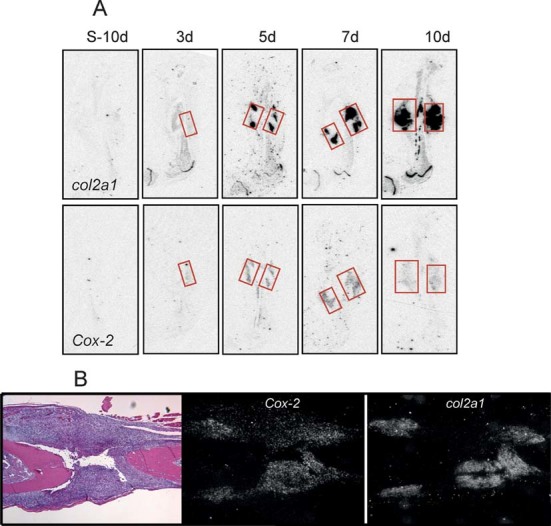
*Cox-2* mRNA is expressed in chondroprogenitor cells in healing fractures. In situ hybridization was performed on fractures obtained from young mice between 3 and 10 days after fracture using murine specific sense and antisense riboprobes for *COX-2* and *col2a1*. Detection was with Kodak Biomax MR X-ray film. *COX-2* expression occurs in regions where early chondrocyte precursors express *col2a1. COX-2* expression subsequently declines as chondrocytes mature (Figure 5A). Microscopic expression was determined in histological sections of day 5 fracture callus and confirms the co-expression of *COX-2* and *col2a1* in early chondroprogenitor cell populations (Figure 5B). S-10d represents use of the sense probe on day 10 tissue sections, which acts as a negative control.

The finding that maximal *COX-2* expression occurs at the onset of chondrogenesis suggests a connection with this process. In situ hybridization and immunohistochemistry were performed to determine whether *COX-2* and *col2a1* were co-expressed in a chondroprogenitor population. Femur sections harvested from young mice between 3 and 10 days after fracture were hybridized to antisense probes specific for *COX-2* and *col2a1,* and X-ray film based auto-radiography was performed (Fig. 4A). The autoradiograph shows abundant expression of *col2a1* in fracture callus and in the distal femoral growth plate. Maximal expression occurred between 7 and 10 days, but lower levels of expression were observed at 3 and 5 days after fracture. *COX-2* hybridization overlapped with areas of *col2a1* expression (Fig. 4A). However, *COX-2* maximal expression occurred at the earlier times (3–7 days) when fracture callus is composed of a less differentiated chondroprogenitor cell population.

To determine the cell populations expressing *COX-2*, emulsion-based autoradiography was performed in day 5 fractures (Fig. 4B). Both *COX-2* and *col2a1* expression was co-localized in populations of chondroprogenitor cells that were transitioning from a flat mesenchymal cell morphology to a rounded chondrocyte in the process of becoming embedded within the matrix. Thus, chondroprogenitors and early chondrocytes are the major source of *COX-2* expression in early fracture repair.

**FIG. 5:**
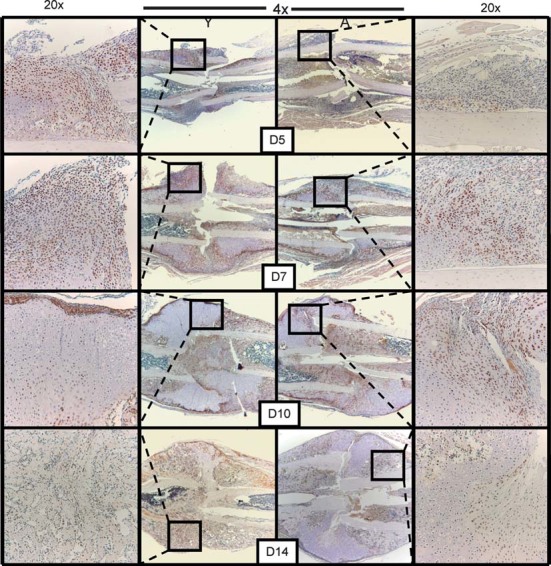
COX-2 protein localizes to chondroprogenitors and the early chondrocyte population. Immunohistochemical staining for COX-2 was performed on fracture calluses from young (left; Y) and aged mice (right; A) between 5 and 14 days. Consistent with mRNA data, COX-2 protein was most abundantly expressed in chondroprogenitors. In young mice at day 10, the COX-2 signal almost completely disappears from cartilage and is limited to the periosteum and osteoblasts. Aged mice have fewer cartilage cells staining positive on days 5 and 7 but have persistent expression in cartilage through days 10 and 14.

To confirm the findings and to examine the relative expression of COX-2 in young and aged mice, immunohistochemical staining for COX-2 was performed on fractures from young and aged mice between 5 and 14 days (Fig. 5). In fractures from young mice, strong COX-2 staining was observed in mesenchymal cells and immature chondrocytes at 5 and 7 days after fracture. By 10 days, COX-2 staining was minimal in cartilage, and at this point, localized primarily to the periosteum and osteoblast population. A different pattern of expression was observed in fractures from aged mice; COX-2 expression was relatively less at 5 and 7 days but persisted in cartilage through 14 days, consistent with the altered gene expression pattern observed in fractures from aged mice.

### COX-2 and RANKL are co-expressed in chondroprogenitor cells in fracture callus

**FIG. 6:**
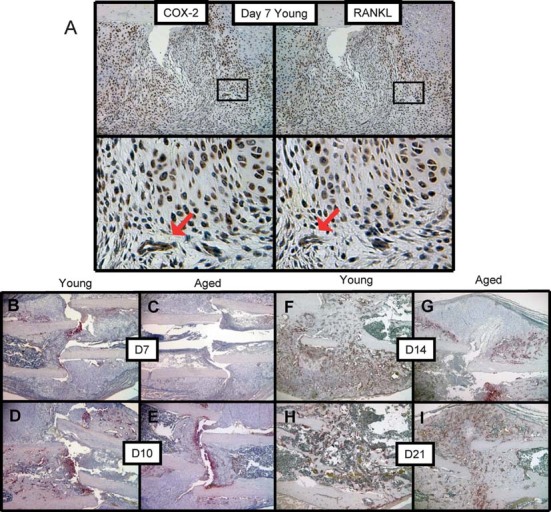
Fractures in aged mice have delayed osteoclast formation. High-power images, ×20 and ×40, show immunostaining using anti-COX-2 and anti-RANKL antibodies in chondroprogenitors and immature chondrocytes at 7 days after fracture. Endothelial cells (arrow) also stained for COX-2 and RANKL (A). Tissues were harvested from young and aged mice at 7, 10, 14, or 21 days after fracture, and sections were stained for TRACP (B-I). The sections from young mice show TRACP^+^ osteoclasts by day 7 along the periosteal surface (B). Osteoclasts increase through day 14 when the entire callus is interspersed with TRACP^+^ osteoclasts (F). By day 21, remodeling is advanced, and the number of osteoclasts is reduced (H). In contrast, osteoclasts do not appear until day 10 in fractures from aged mice, and resorption area appears maximal at 21 days (C, E, G, and I).

It has previously been established that PGE_2_ induces the expression of *RANKL*, a factor essential for the induction of osteoclasts.(20) Immunohistochemistry was performed in 7-day fracture calluses from young mice to examine expression of RANKL and COX-2 because both genes are highly expressed in fractures at that time (Figs. 3G, 3H, and 6A). Fracture sections stained with anti-RANKL and anti-COX-2 show an essentially identical localization of these key signals. Both proteins are localized in immature chondrocytes, osteoblasts, and vascular endothelium but not to hypertrophic chondrocytes.

To evaluate whether the differences in RANKL expression in fracture calluses of young and aged mice was associated with altered osteoclast-mediated bone resorption, TRACP staining was performed on callus tissues between 7 and 21 days and qualitatively assessed (Fig. 6). In young mice, TRACP^+^ osteoclasts were evident by day 7 along the periosteal surface. Abundant remodeling of woven bone was apparent at day 14 throughout the callus. In contrast, the appearance of TRACP^+^ osteoclasts was delayed until 10 days in fractures from aged mice and was not extensive in the callus tissue until 21 days after fracture.

### Local delivery of an EP4R agonist (CP73) rescues the delayed fracture healing phenotype in aged mice

**FIG. 7:**
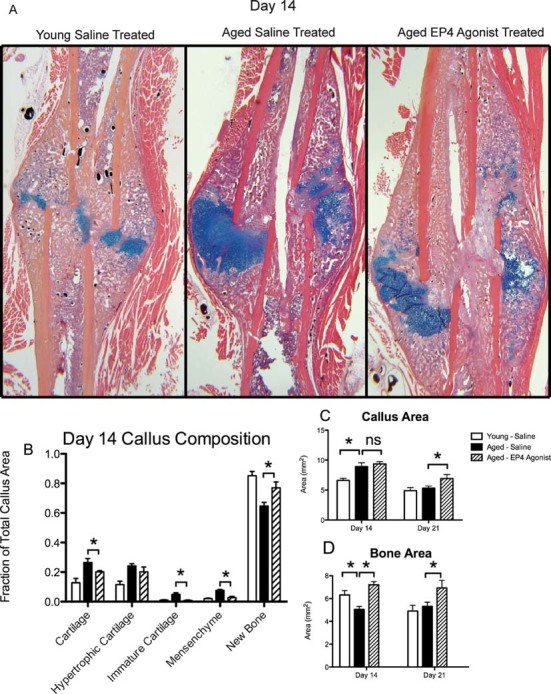
Administration of an EP4 agonist to fractures in aged mice accelerates fracture repair and increase bone formation. Fractures in young mice were injected with vehicle (100 μl twice per day), whereas fractures in aged mice received injection of either vehicle or a selective EP4 agonist (CP73; 20 mg/kg/d). Mice (*n* = 4 per group) were harvested 14 and 21 days after fracture, and tissues were prepared for histology. Representative sections from day 14 are shown in A. Histomorphometry was used to measure the relative populations of undifferentiated mesenchyme, immature cartilage, hypertrophic cartilage, total cartilage, and new bone at day 14 (B). Total callus and bone area was measured on days 14 and 21 (mm^2^) (C and D). CP73 accelerated the completion of endochondral bone formation and resulted in reduced cartilage and increased bone formation compared with the vehicle-treated aged fractures. The anabolic effects of CP73 on bone formation persisted to day 21 in aged animals. These changes compensated for the age-related effects on fracture healing (B-D). Statistical comparisons were performed using ANOVA, and significance is denoted by symbols: *p < 0.05 and **p < 0.005.

The radiographic, histological, and molecular characterization of fractures in aged mice suggests that an early disruption in the progression of healing persists and results in a sustained delay of the entire reparative process. Because *COX-2* is an important early anabolic gene and is markedly reduced in aged fractures, we examined whether the local delivery of the EP4 agonist CP73 could compensate for the delayed fracture repair that occurs in aged mice. CP73 (10 mg/kg/injection) or vehicle was injected into the fractures of aged mice twice daily between 1 and 21 days after fracture and compared with fractures in young mice that received vehicle injections. Tissues harvested after 14 days showed healing in young mice with minimal remaining cartilage and abundant bone formation (Fig. 7A). Fractures in aged mice treated with CP73 had reduced cartilage and increased bone compared with vehicle-treated control fractures (Fig. 7A). Histomorphometry showed that fractures in vehicle-treated young mice were composed of 85% bone, 13% cartilage consisting of 1% immature cartilage, and only 2% undifferentiated mesenchyme. In contrast, fractures in vehicle-treated aged mice were composed of 64% bone, 26% cartilage (5% immature), and 8% undifferentiated mesenchyme. Addition of CP73 increased bone formation to 76% and reduced the composition of cartilage to 20% (1% immature) and 3% undifferentiated mesenchyme (Fig. 7B; *p* < 0.05). Consistent with data shown in Fig. 2, day 14 vehicle-treated fractures in aged mice have increased callus area compared with vehicle-treated young mice (Fig. 7C). Whereas callus area was similar in vehicle and CP73-treated aged mice, CP73 significantly increased bone area, consistent with the more rapid completion of endochondral ossification (Fig. 7D). The total bone area was similar to that observed in the vehicle-treated young mice. Thus, CP73 accelerates the endochondral phase of bone repair in aged mice and results in a pattern of healing that mimics fracture healing in young mice.

In 21-day fractures, untreated (Fig. 2) and vehicle-treated (Fig. 7) fracture calluses in young and aged mice have similar total callus and bone areas (Figs. 2 and 7). CP73 treatment for 21 days resulted in significantly enhanced callus size and bone formation in aged mice compared with the vehicle-treated young and aged mice with fractures. These findings suggest that, in the later phases of fracture repair, CP73 may have additional effects on the osteoblast population that results in increased total bone formation and callus area. Thus, in addition to its early effects of accelerating callus maturation, CP73 also results in increased in callus size and bone formation at later times.

## DISCUSSION

During fracture healing, there is a sequential series of highly linked events that include mesenchymal proliferation, chondrogenesis, chondrocyte maturation and terminal differentiation, vascularization, primary bone formation, and remodeling.(37) Because these events are tightly linked, cellular and molecular alterations that occur early during the healing response may result in an alteration in the timing of subsequent events. Thus, the early cell, tissue, and molecular events that occur immediately after bone injury have particular importance.

The phenotype of fracture repair in aged mice is consistent with altered early gene expression. Compared with young mice, aged mice have delayed formation of callus and chondrogenesis, suggesting a decrease in the rate of proliferation and differentiation of mesenchymal chondroprogenitors. Maximal expression of *col2a1* occurred later, and gene expression persisted for a longer duration. *ColX*, a marker of mature chondrocytes,(38,39) was maximal at 7–10 days in fractures from young mice and at day 10 in fractures at aged mice. Furthermore, fractures in aged mice had reduced peak levels of *colX* expression and expression persisted through 18 days, showing failure to efficiently complete endochondral bone formation. Mature, calcified cartilage acts as a template for bone formation and remodeling. Consistent with the delay and prolongation of the cartilage phase of fracture repair, *osteocalcin* gene peak expression was reduced and expression also persisted in aged callus. Finally, vascularization and fracture union were delayed and the rate of remodeling was reduced in aged fractures. These findings are consistent with previous descriptions of delayed fracture healing in older mice and rats.(7),(40–42)

COX-2 has been shown to be a critical regulator of bone repair in multiple animal models.(19,20),(43–46) *COX-2* expression is rapidly induced in fractures, consistent with our observation of expression in 3 day calluses, with peak expression by 5 days in young mice. COX-2–deficient mice have a fracture healing phenotype that is similar to aged mice, including a slower rate of chondrogenesis, chondrocyte maturation, delayed vascularization, and reduced bone formation. In COX-2^−/−^ mouse fractures, there is persistence of undifferentiated mesenchyme, suggesting that COX-2 is necessary for normal bone and cartilage differentiation in healing tissues.(19)

In vitro studies have confirmed that COX-2 and its metabolite, PGE_2_, enhance the differentiation of MSCs into both cartilage and bone.(19,23,47,48) Addition of PGE_2_ to limb bud MSCs in high-density cultures enhances chondrogenesis.(23) This has been observed in primary avian and murine cultures and mesenchymal cell lines and involves signaling through PKA.(23,47,48) Similarly, PGE_2_ has been shown to stimulate osteoblast differentiation.(19,26) Bone marrow MSC cultures isolated from COX-2^−/−^ mice have reduced osteoblast differentiation. However, this can be compensated by the addition of PGE_2_ to the cultures, suggesting that PGE_2_ is a key metabolite in osteoblast differentiation.(19)

Whereas a role for COX-2 in bone repair has been extensively described, the cell populations responsible for *COX-2* expression in fracture callus have not been defined. In situ hybridization experiments showed co-localization of *COX-2* and *col2a1* in the early chondroprogenitor population. Morphologically, these were flattened fibroblastic-appearing mesenchymal progenitors in the process of becoming embedded within a chondroid matrix. *COX-2* expression was also present in immature chondrocytes but was absent in cells with a hypertrophic phenotype. Interestingly, *COX-2* expression is absent in the growth plate, consistent with lack of a developmental phenotype in COX-2^−/−^ mice. Thus, the expression of *COX-2* seems to be unique to reparative cartilage. *COX-2* expression was also present in the osteoblast population, although gene expression studies suggest that the highest levels of expression were associated with the initial cartilage phases of endochondral bone repair. Whereas prior work has established that osteoblasts express *COX-2*, these are the first experiments to show that cartilage is a major source of *COX-2* during fracture repair. *COX-2* expression was reduced in the fracture callus of aged mice, suggesting that the delayed healing in these mice may be caused by a functional decrease in this enzyme.

PGE-2 is the major metabolite of COX-2 in most tissues and activates one of four receptors, EP1, EP2, EP3, and EP4, that collectively are associated with the protein kinase C (PKC) and PKA signaling pathways. Numerous genes have been shown to be activated by PGE_2_, including *BMP-2* and *RANKL*, two critical factors in bone repair.(20),(49–51) In young fractures, *BMP-2* and *RANKL* both have peak expression during the early cartilage period of fracture repair and are expressed in phase with *COX-2*. EP2 and EP4 agonists have been shown to enhance bone formation in several bone repair models and may have anabolic effects when combined with BMP-2 therapy.(24,26),(52–56) In an ectopic bone formation model, BMP-2 and selective EP4 agonists had a synergistic effect.(56) In rat growth plate chondrocytes, combined activation of EP2 and EP4 receptors resulted in enhanced proliferation and induction of *col2a1* expression.(57) The observation that both anabolic and remodeling genes have peak expressions during the early chondrogenic period of fracture repair further supports the notion that initial cellular and molecular events drive the overall repair process.

Aged mice have reduced and temporally prolonged expressions of both *BMP-2* and *RANKL*. *BMP-2* is regulated by COX-2 and PGE_2_ through EP4.(49) Undifferentiated human MSCs constitutively express more COX-2, PGE_2_, and BMP-2 than mature osteoblasts.(49) When treated with selective COX-2 and EP4 inhibitors, the induction of BMP-2 in these cells was suppressed.(49) Similarly *RANKL* is also regulated by PGE_2_.(20,28,58) Osteoblasts, stromal cells, and fibroblasts all express increased *RANKL* after treatment with PGE_2_.(20,28,58,59) This effect is primarily caused by activation of the EP4 receptor, which activates the PKA signaling pathway. Gain of EP4 function stimulated *RANKL* expression, whereas loss of function prevents *RANKL* expression in PGE_2_-treated cells.(20) *RANKL* has been shown to be expressed in chondrocytes, and our laboratory recently has established that BMP-2 signaling is a potent inducer of *RANKL* expression in chondrocytes.(60) However, direct induction of *RANKL* by PGE_2_ has not been examined in a chondrocyte population. Altogether, regulation of *BMP-2* and *RANKL* by COX-2/PGE_2_ is consistent with an early gene that regulates critical subsequent steps including cell differentiation and subsequent remodeling.

The likely source of the mesenchymal precursor population is the periosteum. Prior work from our laboratory using a murine bone/periosteal cell transplant model clearly established that periosteal cells undergo proliferation and subsequent chondrogenesis in response to injury.(25) In a murine model in which PGE_2_ was delivered to the periosteal surface through an Alzet pump, periosteal bone formation occurred in wildtype and EP1, EP2, and EP3 knockout mice, but not in EP4-deficient mice.(26) This suggests that the periosteal stem cell population is particularly responsive to EP4 signaling. For this reason, we examined whether local delivery of an EP4 receptor agonist could compensate for the reduced rate of fracture repair observed in aged mice.

Local injection of an EP4 agonist to the fracture site of aged mice compensated for the reduced fracture repair observed with aging. Day 14 fractures were selected for detailed analysis because this is the time point in which fractures in young animals are completing the final stages of endochondral ossification and only the final vestiges of hypertrophic cartilage remain. In contrast, aged mice continued to have abundant immature cartilage. Fractures receiving the EP4 agonist had a significant reduction in both immature and hypertrophic cartilage and more efficient completion of endochondral ossification. This effect was readily observed at day 14, when endochondral bone formation is being completed in young mice and fracture union is occurring. As a result, EP4 agonist-treated fractures in aged mice had increased bone formation and developed a histological phenotype that was similar to that observed in young mice. Our gain of function findings are consistent with prior work showing that EP4 knockout mice have delayed endochondral bone formation and fracture healing.(36)

A second finding observed in aged mice with fractures treated with the EP4 agonist was that the total amount of fracture callus was increased in mice treated with CP73 for 21 days. Unlike the observations at 14 days with cartilage maturation, the increase in callus area and bone formation occurred in comparison with both vehicle-treated young and aged mice with fractures. One possible explanation for these findings is that, in the later phases of fracture repair, CP73 may have additional effects on the osteoblast population that results in increased total bone formation and callus area. Thus, once endochondral bone has formed, EP4 may have an additional role in the proliferation, recruitment, or retention of osteoblastic cell populations involved in fracture repair. Prior work has established that EP4 stimulates osteoblast differentiation and matrix production.(26,54,61,62) Furthermore, EP4 knockout mice were observed to have reduced bone with aging and decreased osteoblastogenesis, consistent with an important role in bone formation.(36)

This study focused on the expression pattern of COX-2 in fractures, the reduction in COX-2 expression in fractures in a model of aging, and the potential of COX-2/EP4 gain of function to compensate for and accelerate fracture repair in the setting of aging. The work did not address the issue of whether an EP4 receptor agonist has the potential to stimulate repair in young mice, and this remains an important issue. The molecular events associated with nonunions in young subjects are not understood and it is possible the EP4 receptor gain of function may have a role in promoting normal and delayed fracture repair in young individuals.

The experiments used only a single dose of the EP4 agonist, CP73. CP73 has previously been shown to restore trabecular bone mass and strength in ovariectomized rats.(63,64) Based on the doses used in rats, we chose a daily injection of 20 mg/kg/d, which represents the maximal effects in these animal models. Because the goal of these experiments was to show the concept that a EP4 gain of function can compensate for the reduced rate of fracture healing observed in aged mice, experiments designed to determine the relative potency of different concentrations of CP73 were not completed. Similarly, we did not study the relative potential of other factors, such as BMP-2 or PTH, to compensate for reduced fracture healing in the aging model. PTH and BMP-2 are important and perhaps overlapping or integrated pathways that have been shown to regulate bone repair.(29,51),(65–68) Similar to EP4, the PTH receptor is a G-coupled protein receptor that activates the protein kinase A signaling pathway.(65) Whereas these agents may have an important role in fractures in aging, the current studies focused on the role of EP4 receptor signaling. Future studies will need to determine the relative effectiveness of these and other agents.

Altogether, the experiments showed that the impaired fracture healing with aging involves essentially all stages of the process and suggest that altered expression of early genes involved in fracture repair affect the entire healing cascade. These findings define COX-2/EP4 signaling as an important potential therapeutic target to improve fracture healing in the aging population.
