# Supplementary figures and images for: Reduced COX-2 Expression in Aged Mice Is Associated With Impaired Fracture Healing
Source: J Bone Miner Res. 2008 Oct 13;24(2):251–64. doi: 10.1359/jbmr.081002 (PMC3276605; doi:10.1359/jbmr.081002)

# Supplemental Figure 1

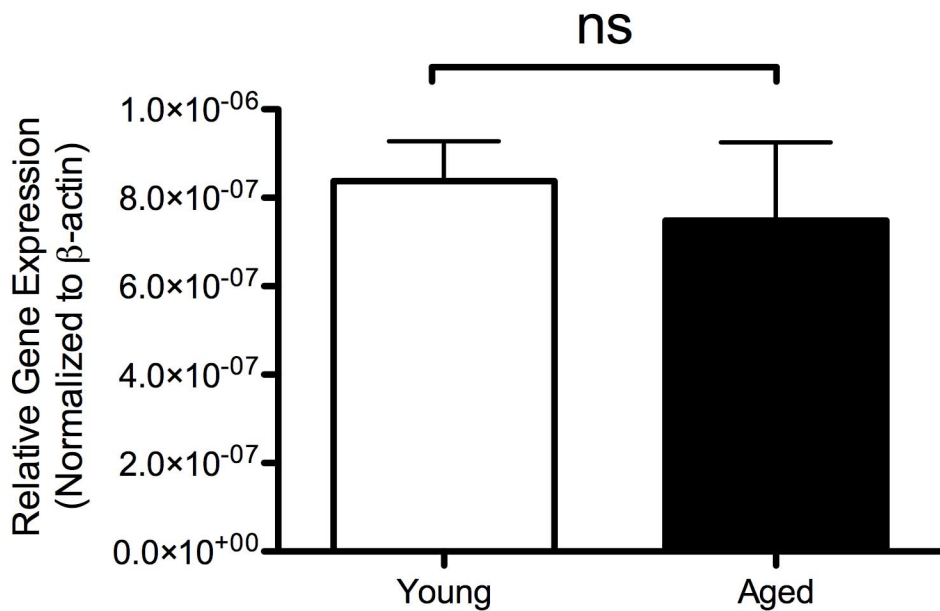

Supplement: Supplementary file 1 [file jbmr0024-0251-SD1.pdf]
